# Development and Application of a KASP-Based SNP Marker System for Fingerprinting, and Genetic Diversity Analysis in Tomato

**DOI:** 10.3390/plants15192944

**Published:** 2026-09-26

**Authors:** Chao Yu, Shupeng Ying, Ruiyi Rong, Chenxu Liu, Jiashun Miao

**Affiliations:** 1 Collaborative Innovation Center for Efficient and Green Production of Agriculture in Mountainous Areas, College of Horticulture, Zhejiang A&F University, Hangzhou 311300, China; yingshupeng777@163.com (S.Y.); 15268002091@163.com (R.R.); 2 Key Laboratory of Vegetable Germplasm Innovation and Quality Breeding in the Province, Institute of Vegetables, Hangzhou, 310021, China; 3 State Key Laboratory for Quality and Safety of Agro-Products, Vegetable Research Institute, Zhejiang Academy of Agricultural Sciences, Hangzhou 310021, China; liuchenxu@zaas.ac.cn; 4 Xianghu Laboratory, Hangzhou 311200, China

**Keywords:** tomato, SNP, KASP, fingerprint map

## Abstract

Tomato is an important vegetable crop worldwide with diverse uses. However, long-term domestication and modern breeding have narrowed the genetic basis of mainstream cultivars, leading to high genetic similarity among varieties. Therefore, developing practical molecular markers through genomic approaches has become an important way to effectively identify and utilize germplasm resources and overcome current breeding bottlenecks. This study aimed to screen high-quality single-nucleotide polymorphism (SNP) loci, develop reliable competitive allele-specific PCR (KASP) markers for genotyping tomato germplasm, and further construct DNA fingerprints and perform genetic diversity analysis, ultimately achieving accurate identification of tomato germplasm. Based on whole-genome resequencing of 241 tomato accessions (inbred lines and wild germplasm), 90 were successfully converted into functional KASP markers. According to polymorphic information content (PIC) and minor allele frequency (MAF), 50 core markers were further screened, with an average PIC of 0.357, an average gene diversity (GD) of 0.46, and an average heterozygosity of 0.063. 16 core SNP markers evenly distributed across 12 chromosomes were optimized, which effectively distinguished all tested accessions. Population structure analysis, principal component analysis (PCA), and phylogenetic analysis indicated that the 241 accessions could be clustered into four major subpopulations, consistent with the results derived from whole-genome SNP data. Finally, a DNA fingerprint of the 241 tomato accessions was constructed based on the 16 core markers. This study provides a robust and cost-effective molecular tool for tomato accession identification, germplasm resource conservation, and genetic diversity assessment.

## 1. Introduction

Tomato (*Solanum lycopersicum* L.) occupies an important position among global vegetable crops. This is not only because of its extremely high economic value, with an annual global production exceeding 188 million tons [1], but also because of its various functions such as fresh consumption, processing, and health care [2]. According to data from the UN’s Food and Agriculture Organization (FAO), China is the world’s biggest grower and consumer of tomatoes, leading in annual production [3]. It plays a vital role in keeping the “vegetable basket” supply and driving farm economic growth [4]. In recent years, with the widespread application of technologies such as molecular breeding, gene editing, and intelligent facility equipment, significant progress has been made in tomato breeding in China. A series of new varieties with notable improvements in yield levels [5], disease resistance [6], storage and transportation tolerance [7], and commercial quality [8] has been successfully developed. However, compared with leading international seed enterprises, there is still a certain gap in flavor [9,10], texture [11], functional nutrient content [12], as well as broad adaptability and stress resistance of tomato varieties in China. Especially under the complex background of global climate change, intensified soil salinization [13], and continuous variation in pathogenic races, the genetic background of existing main cultivars is relatively narrow, making it difficult to fully adapt to diversified planting needs and changes in the consumer market.

Germplasm resources are the foundation of crop genetic improvement. The richness of their genetic diversity is directly related to the potential of breeding innovation and the sustainability of industrial development [14,15]. As a strictly self-pollinating plant, tomato has experienced a significant genetic bottleneck effect during long-term domestication and modern breeding processes, leading to an increasingly narrow genetic basis of current mainstream cultivars and a significant loss of gene pool diversity [16,17,18]. Research indicates that commercial tomato varieties around the globe share a remarkably high degree of genetic similarity, and in China’s Xinjiang region, for instance, major processing tomato varieties share an average genetic similarity coefficient of 0.82 [19]. This high degree of genetic homogenization not only restricts the ability of varieties to cope with climate change and new pests and diseases but also poses the risk of tomato genetic vulnerability caused by a single genetic background [20]. Therefore, the systematic identification, evaluation, and efficient utilization of tomato germplasm resources (especially the genetic variation harbored in their wild relatives) and the development of practical molecular markers (such as DNA fingerprints) using genomic methods [21] have become a key approach to breaking through the current breeding bottlenecks and ensuring future food security [22].

*Solanum lycopersicum* L. was derived from the semi-domesticated cherry tomato (*S. lycopersicum* var. *cerasiforme*) via artificial selection, while the wild progenitor *S. pimpinellifolium* is the closest wild relative of both taxa [23]. Genome sequences of both of the *S. pimpinellifolium* and *Solanum lycopersicum* L. showed only a 0.6% nucleotide divergence [23]. Another member of the same *Lycopersicon* clade, *S. cheesmaniae*, is endemic to the Galápagos islands. Understanding the phylogenetic relationships among these taxa helps clarify how their genetic diversity can be explored and utilized in tomato germplasm. The accurate discrimination of crop varieties forms the foundation for advancing breeding programs and enforcing plant variety rights [24]. In tomato, conventional approaches that depend on morphological and agronomic evaluations are not only time-consuming but also inherently unreliable, as they are easily confounded by environmental factors and subjective judgment [25].

With the development of genomics, molecular markers based on DNA have become the main tool for genetic identification and analysis. Their advantages include the ability to directly reflect genetic composition, high stability, rich polymorphism, and being unaffected by plant developmental stages or growth conditions [26,27]. Studies have found that compared to over 60% of RAPD primers, a few RFLP probes can distinguish between ancient varieties and modern varieties, indicating that RAPD technology has greater potential in detecting subtle genetic changes between closely related genotypes [28,29]. With the widespread adoption of DNA sequencing, Simple Sequence Repeat (SSR) markers developed from resequencing data have demonstrated considerable utility. They are not only effective for germplasm identification but are also efficiently applied in assessing the genetic purity of hybrid seeds [30,31].

The tomato genome (~900 Mb, 12 chromosomes) was first sequenced in 2012 by the Tomato Genome Consortium using the processing cultivar ‘Heinz 1706’ as the reference, producing a ~760 Mb assembly [32]. Hosmani et al. [33] updated the same genotype to SL4.0, yielding a 782.5 Mb assembly. More recently, a substantially improved reference was generated by integrating PacBio long-read sequencing with Hi-C chromatin interaction maps, resulting in a more contiguous and complete genome assembly [34]. Owing to their high genomic density, abundance, and codominant inheritance, single-nucleotide polymorphisms (SNPs) have become the standard third-generation molecular marker and the marker system recommended by UPOV for plant variety protection [35,36]. The competitive allele-specific PCR (KASP) assay, a medium-throughput and cost-effective genotyping platform, has been widely adopted to leverage these advantages, with demonstrated efficacy in large-scale genotyping and variety identification for crops such as cabbage, radish, broccoli, watermelon, and pepper [37,38,39,40,41]. However, applying this approach systematically to tomato lags behind that of other crops. There is a distinct lack of developed, genome-wide SNP fingerprinting systems that encompass the full pipeline from SNP discovery to KASP assay design and validation.

However, although molecular markers have been widely employed in tomato breeding programs, reports on the application of KASP-based SNP markers in tomato germplasm research remain relatively limited. Existing studies have predominantly focused on trait-specific KASP markers [42,43,44,45,46,47], and the germplasm studied primarily consists of commercial tomato cultivars [48]. Meanwhile, Xu et al. [49] successfully established a SNP fingerprint for 418 tomato accessions using restriction-site associated DNA sequencing; however, the resulting markers are inherently constrained by restriction enzyme recognition sites. This limitation may lead to uneven genome coverage and increased difficulty in ensuring flanking sequence conservation during KASP primer design. Furthermore, the limited coverage of tomato materials in previous studies restricts the broad application of core markers. Consequently, developing SNP markers with better polymorphism, operational convenience, and easy detection is essential for saving time and effort in large-scale tomato sample analyses. To address these limitations, we assembled a germplasm panel comprising 241 accessions that are exclusively inbred (self-pollinated) lines or wild accessions; no F1 hybrid accessions were included. We employed whole-genome resequencing to mine high-quality SNPs from this panel, enabling the development of a candidate core KASP-SNP marker set specifically designed for comprehensive fingerprinting, genetic diversity, and population structure analysis.

## 2. Results

### 2.1. Development and Characterization of KASP

Based on whole-genome resequencing of 241 tomato accessions (inbred lines and wild germplasm), 106 SNP loci were randomly selected to achieve uniform coverage across the 12 tomato chromosomes, of which 90 were successfully converted into functional KASP markers. Based on polymorphism information content and minor allele frequency, 50 core markers were retained for subsequent research (Supplementary Tables S2–S4). These markers were reliably genotyped and distributed across the 12 chromosomes (Figure 1A). Analysis of these 50 SNPs identified 11 distinct nucleotide substitution types. The most frequent genotypes were A/T (30%), C/T (22%), and T/A (14%) (Figure 1B). The diversity and proportions of these substitution types reflect the natural spectrum of single-nucleotide variation in the genome. To assess their quality, we calculated PIC. Across 241 materials, the 50 markers had an average PIC of 0.357, which falls within the medium-to-high range and confirms their suitability (Figure 2A). Additional genetic diversity analysis further supported the markers’ stability for use in subsequent studies.

For these markers, minor allele frequency (MAF) values spanned from 0.249 to 0.493, averaging 0.366 (Figure 2B). The average gene diversity was 0.46, indicating substantial genetic polymorphism. In contrast, the average heterozygosity was only 0.063—a pattern typical of self-pollinating crops such as tomato (Figure 2C,D). Based on these combined results, the 50 candidate core SNPs form a reliable marker set for genetic diversity analysis in tomato germplasm.

### 2.2. Optimization of Core SNP Markers

To optimize the molecular marker system for tomato accessions identification, we manually selected the optimal marker combinations from 50 candidate core SNPs, ultimately selecting a core set of 16 markers, uniformly distributed across the 12 chromosomes (Figure 3A). The number of distinguishable samples rose sharply with the first few markers added, showing that a small number of high-quality SNPs can substantially improve identification efficiency. After approximately 10 markers, the curve flattened, indicating diminishing returns in sample discrimination from additional markers (Figure 3B).

### 2.3. Genetic Diversity and Population Structure Analysis of Tomato Germplasm

The phylogenetic tree based on 16 SNP markers resolved the 241 tomato accessions into distinct genetic clusters, corresponding largely to their taxonomic groups (Figure 4). Cultivated tomato (*S. lycopersicum*, blue group) formed a large, cohesive clade, indicating a highly convergent genetic background. Cherry tomato (*S. lycopersicum* var. *cerasiforme*, green group) and currant tomato (*S. pimpinellifolium*, purple group) did not form separate monophyletic clusters. Instead, individuals from these groups were distributed across multiple branches within or adjacent to the cultivated tomato clade. This pattern reflects the known close genetic relationships among these groups. The single accession of *S. cheesmaniae* (red) was placed near the periphery of the main cultivated tomato branch, showing a degree of genetic affinity to some cultivated materials while maintaining some separation. It should be noted that this observation is based on a single accession and cannot be generalized to the species level.

Principal component analysis (PCA) based on 16 SNP markers revealed the genetic structure among the 241 tomato accessions. The first two principal components, PC1 and PC2, explained 19.5% and 14.1% of the total genetic variation, respectively (Figure 5). In the two-dimensional plot, cultivated tomatoes (*S. lycopersicum*, blue group) were subdivided into two major clusters. Cherry tomato (*S. lycopersicum* var. *cerasiforme*, green group) showed separation from cultivated tomatoes but overlap with currant tomato (*S. pimpinellifolium*, purple group). The single accession of *S. cheesmaniae* was positioned at the margin of the main cultivated clusters in the PCA space, displaying clear genetic distinction from the core groups. The apparent difference in its placement between the phylogenetic tree and the PCA reflects the distinct assumptions of hierarchical clustering versus variance-based ordination, rather than conflicting signals in the data.

Population structure analysis based on the 16 core SNP markers revealed hierarchical genetic differentiation among the 241 tomato accessions (Figure 6). Cross-validation analysis identified K = 4 as the most supported clustering solution (Supplementary Figure S2). At K = 2, the accessions were broadly separated into two major ancestry components. Increasing K to 3 further resolved differentiation among cultivated tomato, cherry tomato (*S. lycopersicum* var. *cerasiforme*), and currant tomato (*S. pimpinellifolium*) groups. At K = 4, the inferred ancestry components showed clearer differentiation and were largely consistent with PCA and phylogenetic analyses, supporting the presence of major genetic subdivisions within the germplasm panel. Higher K values (e.g., K = 5 and K = 6) further subdivided some groups but resulted in more complex ancestry patterns with limited additional biological resolution. Therefore, K = 4 was considered the most biologically interpretable population structure for this dataset.

### 2.4. Construction of a SNP Fingerprint Map

Using these 16 core SNP markers, we constructed a DNA fingerprint map for the 241 tomato accessions (Figure 7). The map confirmed that all 16 core markers were polymorphic across the population, displaying both homozygous and heterozygous genotypes. Considerable variation in allele frequencies among loci underscored their discriminatory power. With only occasional missing calls, each accession possessed a unique genotype combination, creating a distinct molecular fingerprint that clearly differentiated germplasms.

Clustering patterns at the top of the map grouped accessions with similar genetic backgrounds; their genotype distributions aligned closely. These patterns matched earlier PCA and population structure results, reinforcing the effectiveness of the core marker set. Fingerprint comparisons identified specific material pairs, such as C60 and C431, C384 and C385, C368 and C73, C18 and C437, C2 and C24, C11 and C21, C214 and C473, C137 and C295, C297 and C357, C407 and C441, C192 and C279, C211 and C547, and C449 and C568, that differed at just one SNP locus or only by a missing genotype. Such differences should be interpreted cautiously, as they do not provide strong evidence of genetic distinctness. These cases may represent different batches of the same accession, misnamed materials, or closely related sister lines, and warrant further validation with additional markers or independent genotyping. In contrast, pairs like C79 and C179, C221 and C406, C337 and C97, C393 and C575, and C100 and C444 differed at two or more SNP loci, confirming clear genetic distinction. In practice, the 16 core KASP markers provide a high-resolution but preliminary tool for distinguishing tomato accessions with minimal genotyping effort and cost. Its stability and standardization support applications in accession authentication, germplasm management, intellectual property protection, and hybrid seed purity testing.

## 3. Discussion

Given that the assessment of tomato germplasm diversity can vary with different genetic materials and that phenotypic traits are often influenced by both genotype and environment, molecular markers offer a more robust and precise tool for analyzing crop genetic diversity [7]. SNP-based KASP assays now serve as a mainstream platform for crop genotyping and variety identification, offering genome-wide coverage, high stability, and cost-effectiveness [50,51,52]. Despite its advantages, comprehensive tomato fingerprint databases that integrate SNP loci, KASP technology, and genetic diversity analysis are scarce. Research utilizing SNP markers to analyze tomato germplasm remains in its early stages. For instance, Xu et al. [49] developed and evaluated a SNP fingerprint for 418 tomato accessions using restriction-site associated DNA sequencing (ddRAD). The resulting markers are inherently constrained by restriction enzyme recognition sites. This limitation may lead to uneven genome coverage and increased difficulty in ensuring flanking sequence conservation during KASP primer design [53]. Given the inherently limited genome coverage of reduced-representation sequencing and the rapid decline in sequencing costs, whole-genome resequencing has emerged as the preferred approach, offering superior efficiency in identifying novel loci [54]. In addition, the germplasm representation in earlier studies has been biased toward elite breeding lines [46,47,55] or commercial F_1_ hybrids which are highly heterozygous, limiting the broader applicability of the identified core markers across diverse genetic backgrounds. The coverage of tomato materials in previous studies still exhibits certain limitations. Unlike these earlier studies, our germplasm panel consisted of 241 inbred lines and wild accessions, with no F_1_ hybrids. These accessions covered a wide range of tomato types, geographic origins, and trait characteristics. We used whole-genome resequencing to identify high-quality SNPs, and then developed a set of polymorphic KASP markers for fingerprinting, genetic diversity, and population structure analysis.

In previous studies, Xu et al. [49] used ddRAD sequencing to conduct a comprehensive analysis of genetic diversity and population structure across 418 globally representative tomato cultivars, establishing a streamlined DNA fingerprinting system that required only 15 core SNPs for cost-effective variety identification [49]. In a separate effort, researchers constructed a core collection comprising 137 accessions from a pool of 484 tomato germplasm resources by integrating phenotypic traits and SNP marker data. Their work identified an optimal strategy (employing Mahalanobis distance with a 10% sampling ratio) for developing a representative core set [47]. Moreover, a systematic comparative evaluation between SNP fingerprinting and the conventional Distinctness, Uniformity, and Stability (DUS) test confirmed a high degree of concordance between the two methods. This comparison facilitated the development of a minimized diagnostic panel consisting of 16 core SNPs and 18 core DUS traits for efficient variety discrimination [46]. In the present study, based on PIC and MAF, 50 core markers were further screened. From these, 16 core SNP markers evenly distributed across 12 chromosomes were optimized, which effectively distinguished all tested accessions. This marker set developed is distinct from previously reported SNP panels [47,49,56] and represents a complementary rather than a redundant resource (Supplementary Table S7). We note that a small proportion of our materials exhibited residual heterozygosity, a common phenomenon in advanced-generation lines, which does not undermine the fundamental distinction from the fully heterozygous F_1_ hybrids used in previous studies. This distinction is critical, as markers optimal for heterozygotes may not be equally effective for near-homozygous lines [46].

In our study, investigations based on this marker set, including population structure, principal component, and phylogenetic analyses (Figure 4, Figure 5 and Figure 6), grouped the tested materials into clusters that were broadly consistent with their genetic backgrounds and fruit weight (Supplementary Table S1), demonstrating the practical utility of this marker panel for accession classification and identification. These findings are consistent with those reported in previous studies [47,49,56]. And the patterns of genetic diversity observed among the clusters are further supported by the genome-wide dataset, confirming the reliability of the 16 core SNP markers (Supplementary Figure S3). At the same time, the 16 core markers were selected for high PIC, even chromosome distribution, and reliable genotyping, and their ability to distinguish accessions was further validated using 272 independent external tomato accessions (Supplementary Figures S4 and S5). The results demonstrated that the 16-SNP panel was able to distinguish most of the additional accessions, supporting its practical value as a rapid and cost-effective fingerprinting tool. Meanwhile, we acknowledge that several external accessions shared identical fingerprints based on the 16 SNP markers. This result is expected because the discriminatory power of a fingerprinting system is influenced by both marker number and the genetic diversity of the tested population. A larger or genetically distinct germplasm collection may therefore require additional markers or complementary information to achieve complete discrimination. We also note that a leave-one-group-out analysis was not done here. Such an analysis could help determine marker redundancy and the minimum number of markers needed to discriminate all accessions apart. Furthermore, we also acknowledge that differences based on only one SNP or missing data are not robust evidence of distinctness, and that probability of identity and error tolerance were not assessed here. These metrics remain important for a practical fingerprinting system. Future work with larger, independent sample sets could apply these methods to further evaluate the robustness and efficiency of the marker set. The expanded germplasm collections used in such efforts could also serve as a foundation for systematic identification of species- or variety-specific alleles, which we consider an important direction for future studies. Given the risk that long-term directional selection targeting specific phenotypes (e.g., fruit shape) may lead to genetic narrowing of the breeding gene pool, such methodological improvements are particularly warranted. To mitigate this risk, future tomato breeding strategies should shift from phenotype-dependent selection toward systematic selection based on favorable genotypes, thereby actively broadening and preserving the genetic basis of breeding populations [57,58].

The construction of DNA fingerprinting maps for tomato varieties offers a reliable legal and technical foundation for variety protection and market oversight, such as addressing the issue of “multiple names for a single variety” [59]. By refining the core SNP marker set, it is possible to lower costs while enhancing the efficiency of variety purity assessment, thereby providing a practical solution for seed quality monitoring [49]. Further integration of population genetic analysis and genome-wide association studies enables the discovery of functional markers associated with key agronomic traits, advancing tomato breeding from an experience-driven practice toward a precision design-based approach [60,61]. With the adoption of new breeding techniques such as gene editing and marker-assisted introgression, the number of essentially derived varieties (EDVs) is increasing in tomato. Although the 16-core SNP panel showed high discriminatory power within the evaluated tomato germplasm collection, it may have limited resolution for identifying essentially derived varieties (EDVs), particularly when the derivative differs from the original variety by only one or a few genomic regions. With the increasing application of modern breeding approaches, including genome editing and targeted introgression, EDV identification will require tailored strategies that combine genome-wide similarity assessment with trait-associated diagnostic markers. Future development of modular SNP panels with additional informative loci may provide a more flexible framework for distinguishing closely related materials.

In summary, this study used whole-genome resequencing data to build an efficient SNP fingerprinting system based on KASP technology, and also constructed a core germplasm collection for tomato. This system offers reliable technical support and a theoretical framework for accurately identifying tomato accessions, analyzing genetic relationships, and conserving and using germplasm resources more efficiently. In the future, as more tomato germplasm is collected and sequenced, functional SNP markers closely linked to important traits (such as disease resistance, stress tolerance, and fruit quality) can be further integrated and developed. This will allow the fingerprinting platform to be continuously improved, helping to drive the sustainable development of molecular design breeding and the tomato industry.

## 4. Materials and Methods

### 4.1. Plant Materials and DNA Extraction

241 representative tomato (*Solanum lycopersicum* L.) germplasm accessions were used, including 145 *S. lycopersicum* L. (advanced inbred lines; 128 from Zhejiang Academy of Agricultural Sciences, 18 from Zhejiang University, 1 from United States Department of Agricultural (USDA)), 55 *S. lycopersicum* L. var. *cerasiforme* (cherry tomato, advanced inbred lines; 34 from USDA, 20 from Zhejiang Academy of Agricultural Sciences, 1 from Zhejiang University), 40 *S. pimpinellifolium* L. (wild; 31 from USDA, 9 from Zhejiang Academy of Agricultural Sciences), and 1 *S. cheesmaniae* (L. Riley) Fosberg (wild) from USDA (Supplementary Table S1). Plants were cultivated in an artificial climate-controlled growth chamber under the following conditions: a 16-h light/8-h dark photoperiod, a day/night temperature regime of 26 °C/18 °C, and a relative humidity of 50–60%. Young leaf tissues were collected, immediately frozen in liquid nitrogen, and stored at −80 °C until use.

Genomic DNA was extracted from approximately 100 mg of fresh leaf tissue using a modified CTAB method [28,46]. Genomic DNA was extracted from leaf samples using the CTAB method. Briefly, ground leaf powder was lysed in 2× CTAB buffer at 65 °C for 30 min, followed by purification with chloroform: isoamyl alcohol (24:1) and precipitation with isopropanol. The DNA pellet was washed, resuspended in TE buffer, and assessed for quality and concentration using a NanoDrop 2000 spectrophotometer (Thermo Fisher Scientific, USA) and 1.0% agarose gel electrophoresis. Qualified DNA samples were normalized to 20 ng/µL for downstream analysis.

### 4.2. Development of SNP-Based KASP Markers

High-quality, genome-wide SNP sites were identified from whole-genome resequencing data of the 241 tomato accessions. Reads were aligned to the tomato reference genome (SL4.0, https://solgenomics.net/ftp/tomato_genome/Heinz1706/assembly/build_4.00/ (accessed on 2 February 2026)). Raw sequencing data quality was assessed with FastQC, and low-quality reads, adapter sequences, and reads with excessive ambiguous bases were removed using Fastp (v0.23.1) [62]. Clean reads were aligned to the reference genome using BWA-MEM (v0.7.17) [63]. Resulting SAM files were converted to BAM format, sorted, and deduplicated with Samtools. SNP calling was performed using the Genome Analysis Toolkit (GATK) (v4.2.6.1) [64]. The pipeline included base quality score recalibration and InDel realignment, followed by variant calling with HaplotypeCaller. An initial filter was applied to remove low-confidence SNPs based on GATK recommended thresholds (QD < 2.0, MQ < 40.0, MQRankSum < −12.5, ReadPosRankSum < −8.0). To obtain SNPs suitable for marker development, further filtering ensured site uniqueness and robust population genetics parameters. A custom Python 3.9 script verified that no other variants (e.g., InDels) were present within 100 bp upstream or downstream of each SNP. VCFtools (v0.1.16) was then used to apply filters for minimum allele count (≥3), minimum allele frequency (≥0.05), average sequencing depth (≥4), site quality (≥30), and missing rate (≤20%) [65]. This stringent pipeline yielded 8,668,967 high-quality SNP sites for subsequent genetic analysis. From the resequencing data, 600 SNP loci were selected based on the following criteria: (1) no other variants within 100 bp flanking each locus; (2) PIC > 0.3; (3) minor allele frequency (MAF) > 0.1; and (4) uniform distribution across chromosomes. Sequences flanking the candidate SNPs (100 bp on each side) were extracted for KASP marker design. For each target locus, two allele-specific primers and one common primer were designed. For each selected locus, two allele-specific forward primers (with FAM or HEX tails) and one common reverse primer were designed using PrimerPicker (LGC Biosearch Technologies). Primer design targeted amplicons of 80–120 bp, with annealing temperatures between 55–62 °C and GC content below 60%. All primers were commercially synthesized. Genotyping reactions were set up in a 1.6 µL volume containing 0.8 µL of 2× KASP Master Mix, 0.016 µL of primer mix (~12 nM final concentration), and 0.8 µL of template DNA (20 ng/µL). Each 96-well plate included two no-template controls (NTCs) containing ddH_2_O. Amplification was performed on an IntelliQube system with the following program: 94 °C for 15 min; 10 touchdown cycles of 94 °C for 20 s and 61–55 °C for 60 s (−0.6 °C per cycle); then 30 cycles of 94 °C for 20 s and 55 °C for 60 s. Fluorescence signals were read on the same instrument, and scatter plots of the results were generated for analysis. For validation, 20 samples were randomly selected from the re-sequenced accessions and assayed with the successfully designed KASP primers, each in triplicate independent PCR reactions to assess reproducibility. Polymorphic primers with clear clustering, high genotyping accuracy, and good reproducibility were selected as core primers. Fifteen KASP markers failed to genotype successfully or produced indistinct results, and one showed only a single genotype with no polymorphism.

### 4.3. SNP Marker Selection and Fingerprinting Analysis

To develop a minimal SNP-based fingerprinting panel, genome-wide polymorphic SNP markers were evaluated based on their discriminatory power and genomic distribution. Genotype data were standardized by converting reciprocal heterozygous genotypes (e.g., A/T and T/A) into a consistent format. Missing genotypes were treated as missing data during marker evaluation.

Core SNP markers were selected using an iterative discrimination-based marker selection strategy. Briefly, candidate SNPs were sequentially added according to their ability to maximize the number of unique multilocus genotype profiles among all accessions. At each iteration, the SNP generating the greatest increase in the number of distinguishable genotype combinations was selected. To ensure genome-wide representativeness, a chromosome coverage constraint was implemented by requiring at least one informative SNP marker from each chromosome. Marker selection was terminated when all accessions were uniquely identified or when no further improvement in discrimination efficiency was achieved.

The discriminatory capacity of the selected SNP panel was evaluated using Simpson’s diversity index: $D=1-\sum_{1}^{i}p_{i}^{2}$, where *p* represents the frequency of each multilocus genotype class. Simpson’s index has been widely applied for evaluating diversity and discrimination efficiency in genetic identification systems [66,67]. The genotype fingerprints generated by the selected SNP panel were visualized using heatmap representations. The physical distribution of core SNP markers across chromosomes was plotted according to their genomic positions.

### 4.4. Data Analysis

Genotyping data from KASP assays were collated and formatted in Microsoft Excel 2021. Homozygous SNP calls were recorded as A/A, T/T, G/G, or C/C, and heterozygous calls were denoted with a slash (e.g., A/C). Genetic diversity parameters—PIC, MAF, GD, and He—were computed for all valid markers using PowerMarker (v3.25). GD = 1 − Σp_i_^2^, where p_i_ is the frequency of the i-th allele. PIC = 1 − Σp_i_^2^ − Σ_i<j_ 2p_i_^2^p_j_^2^, where p_i_ and p_j_ are the frequencies of the i-th and j-th alleles, respectively, and n is the number of alleles at the locus. A pairwise genetic distance matrix derived from the genotype data served as the basis for constructing a neighbor-joining (NJ) phylogenetic tree, applying Nei’s 1983 algorithm [68]. The tree was visualized with MEGA-X and further edited on the Interactive Tree Of Life (iTOL) website.

PCA was conducted on the genotype data using TASSEL (v5.1), and results were plotted with R. A fingerprint heatmap was generated in R version 4.4.1 (2024-06-14) from this marker set, where each row represents an SNP locus and each column a sample. Colors denote homozygous genotypes: AA (blue), CC (orange), GG (yellow), TT (purple); heterozygous genotypes (green); and missing data (gray).

Genetic relationships among accessions were assessed using principal component analysis (PCA), neighbor-joining (NJ) phylogenetic reconstruction, and model-based ancestry estimation. PCA was performed using standardized genotype matrices to summarize major patterns of genetic variation among accessions [69]. Pairwise genetic distances were calculated from SNP genotype data, and a neighbor-joining tree was constructed using the NJ algorithm [70]. Phylogenetic visualization was performed using the R package ggtree [71].

Population structure was inferred using the sparse non-negative matrix factorization (sNMF) method implemented in the R package LEA [72]. The sNMF algorithm estimates individual ancestry coefficients while accounting for linkage disequilibrium and has been widely used for population structure analysis of plant genetic resources. The number of ancestral populations (K) was evaluated from K = 1 to 10, and the optimal K value was determined based on cross-entropy criteria [73].

## Figures and Tables

**Figure 1:**
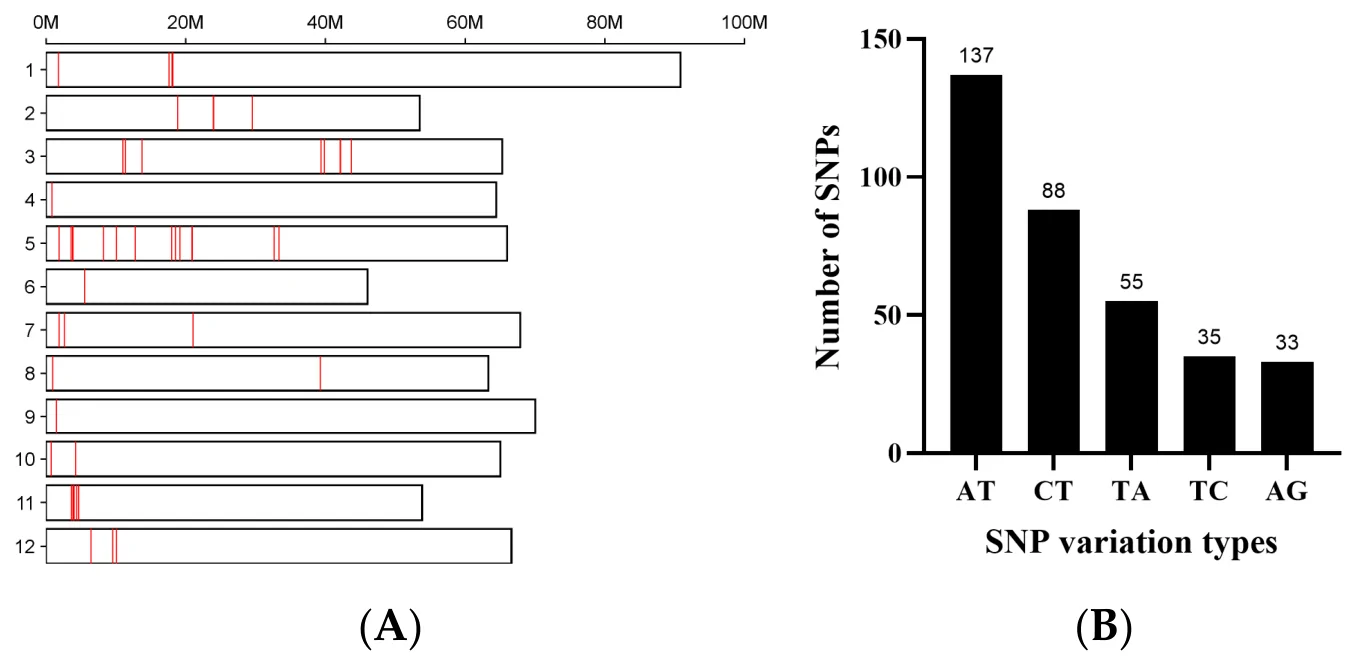
SNP screening and statistics. (**A**) Development of 50 high-quality SNPs. (**B**) Genotype statistics for the most commonly identified variants.

**Figure 2:**
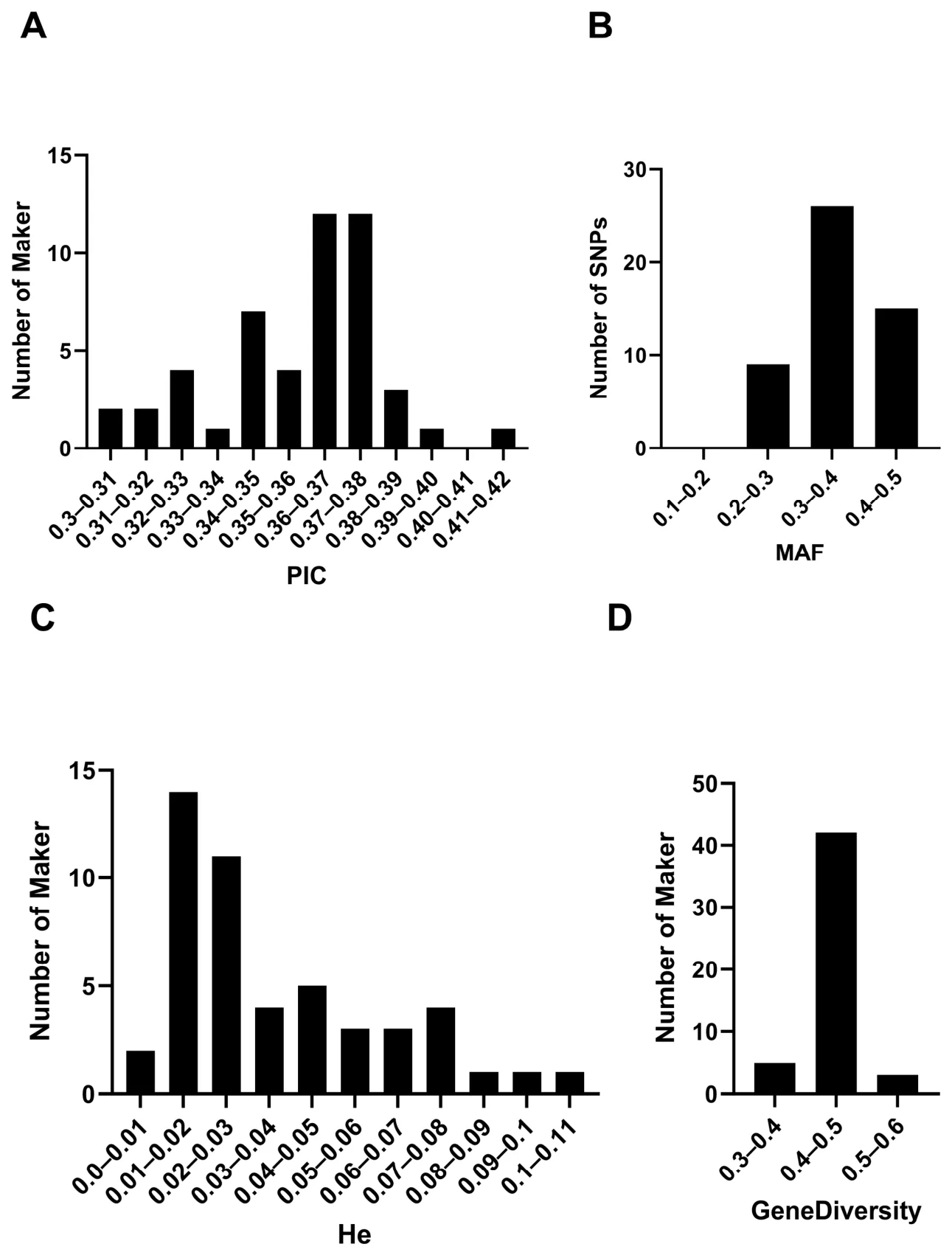
Genetics analysis of 50 candidate core markers in 241 tomato materials. (**A**) Polymorphic information content (PIC); (**B**) Minor allele frequency (MAF); (**C**) Heterozygosity (He); (**D**) Gene diversity (GD).

**Figure 3:**
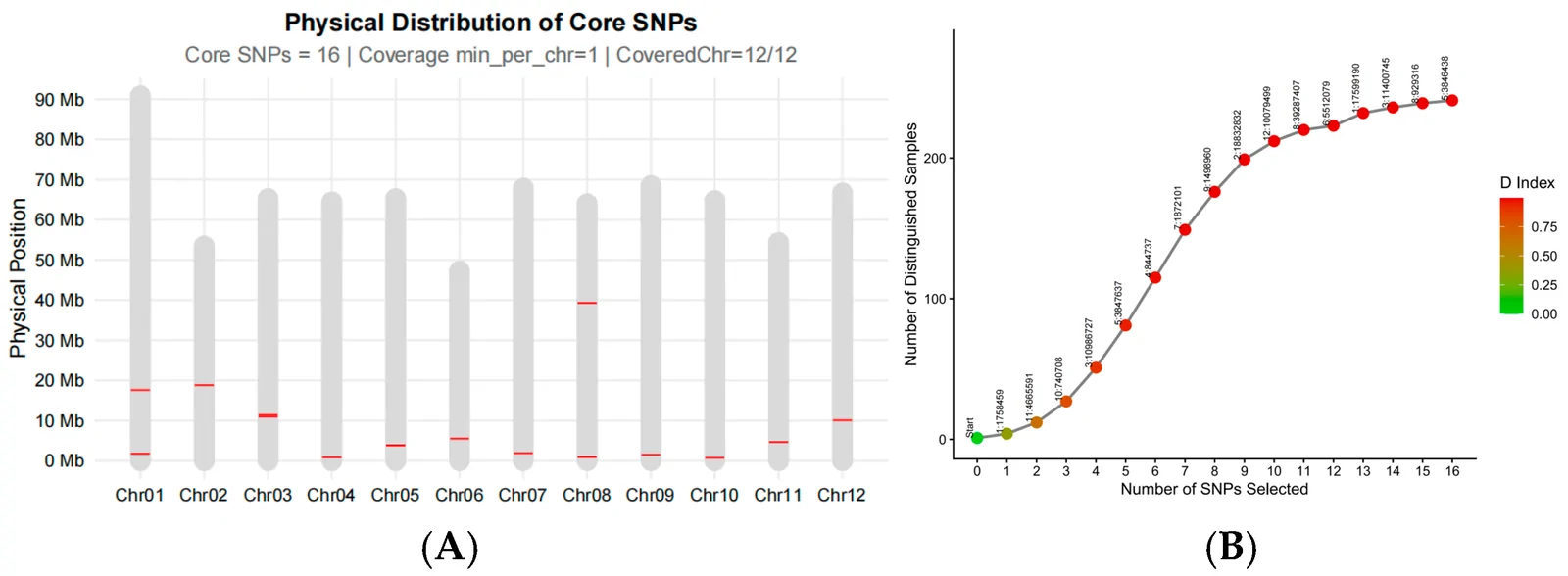
Distribution of 16 core markers on tomato (**A**) and Identification efficiency of the combined SNP markers (**B**).

**Figure 4:**
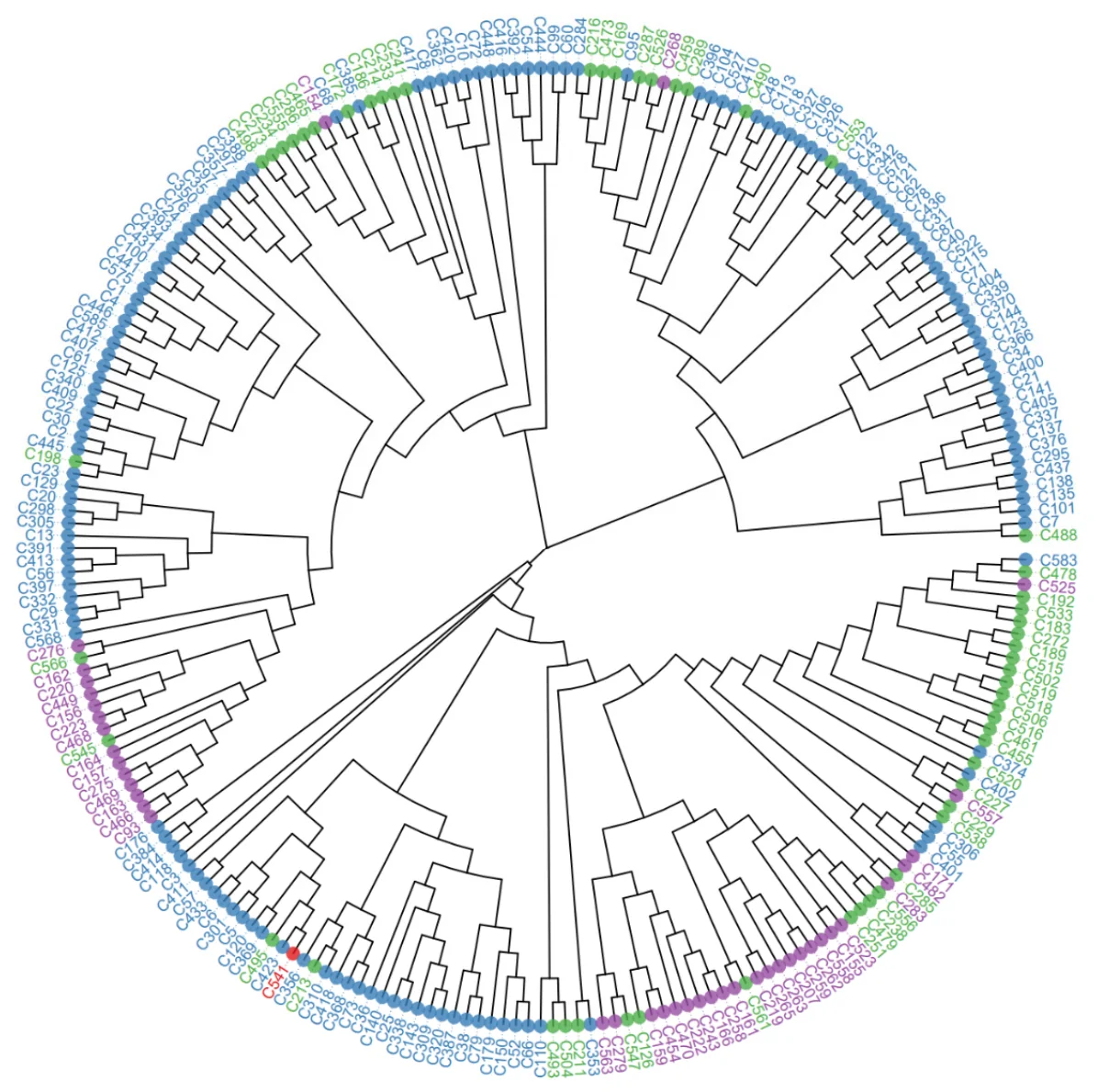
Phylogenetic tree of the 241 tomato accessions based on the 16 core KASP markers. Note: Blue-highlighted accessions represent *S. lycopersicum* L.; green-highlighted accessions represent *S. lycopersicum* var. *cerasiforme*; purple-highlighted accessions represent *S. pimpinellifolium*; red-highlighted accessions represent *S. cheesmaniae*.

**Figure 5:**
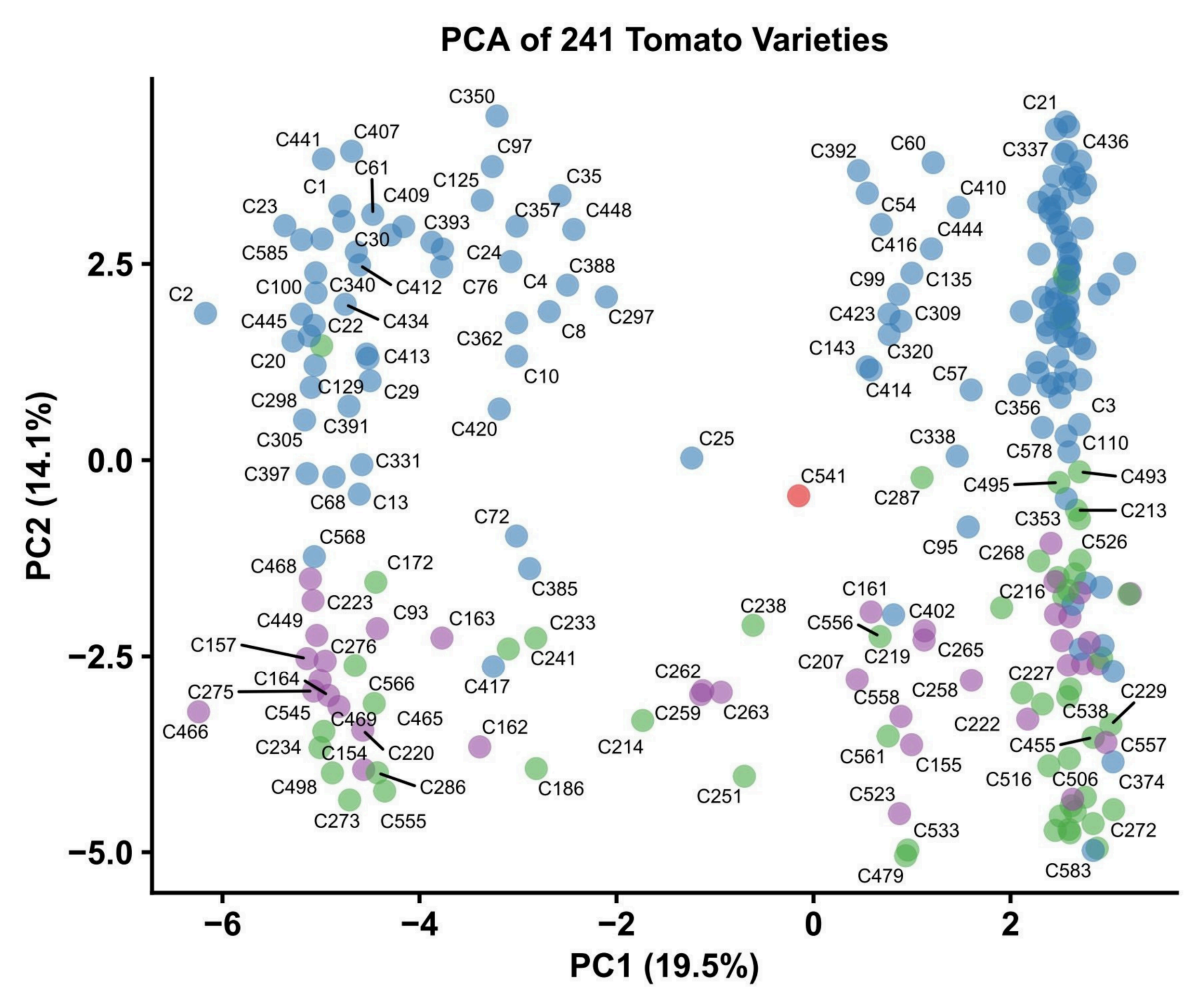
Principal Component Analysis (PCA) of the 241 tomato accessions. Note: Blue-highlighted accessions represent *S. lycopersicum* L.; green-highlighted accessions represent *S. lycopersicum* var. *cerasiforme*; purple-highlighted accessions represent *S. pimpinellifolium*; red-highlighted accessions represent *S. cheesmaniae*.

**Figure 6:**
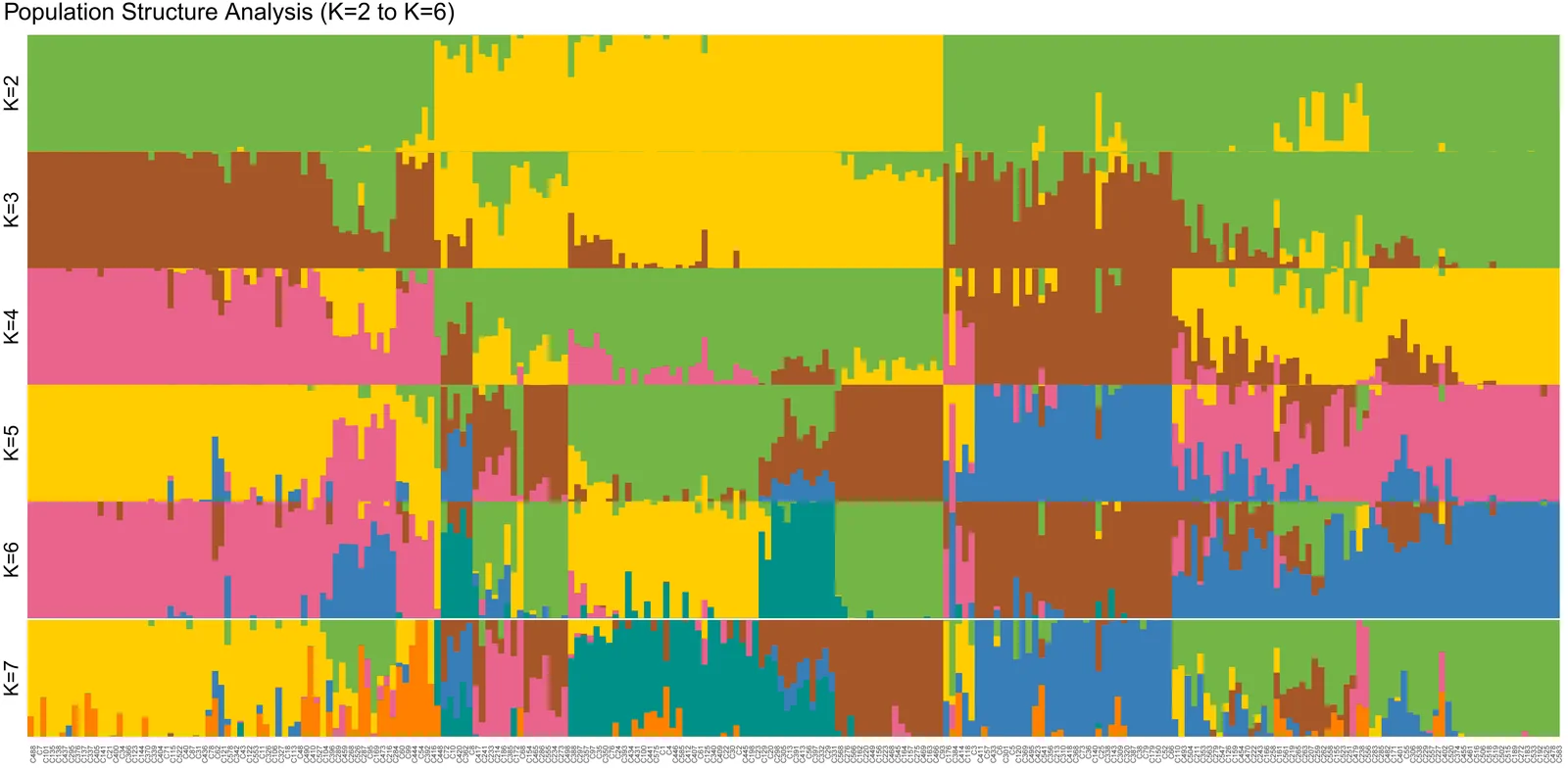
Population structure of the 241 tomato germplasm accessions. The analysis was performed for K = 2–7, where K represents the assumed number of genetic clusters. Each vertical bar represents one tomato accession, and the colored segments within each bar indicate the estimated ancestry proportions assigned to the inferred genetic clusters. Accession codes are shown below the bars.

**Figure 7:**
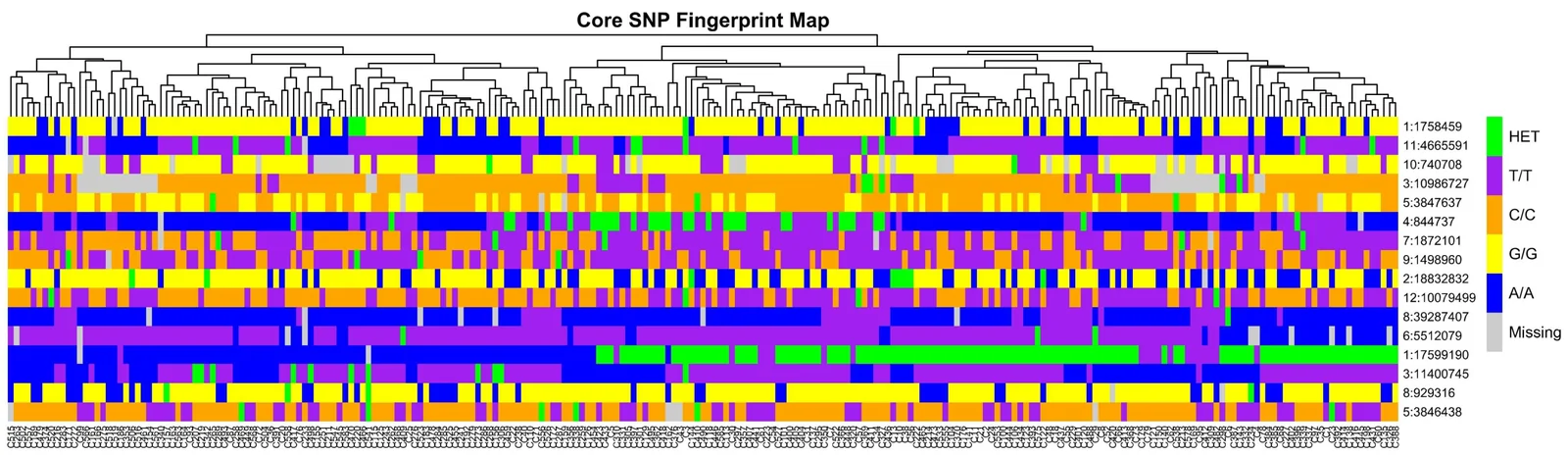
DNA fingerprinting of 241 tomato germplasm accessions based on the 16 core SNP markers. Each column represents one tomato accession, and each row represents one of the 16 core SNP markers. The colored cells indicate the genotype of each accession at the corresponding SNP locus: T/T, C/C, G/G, and A/A represent the corresponding homozygous genotypes, HET represents a heterozygous genotype, and Missing indicates a missing genotype. Accession codes are shown below the fingerprint map, and SNP marker identifiers are shown on the right. The dendrogram at the top represents the clustering of accessions based on their SNP genotype profiles.

## Data Availability

The raw data of genome resequencing is uploaded to National Genomics Data Center (NGDC), and the accession number is CRA044783. Data sharing is available upon request.

## Supplementary Materials

The following supporting information can be downloaded at: https://www.mdpi.com/article/10.3390/plants15192944/s1. Table S1: Quality assessment of resequencing data; Table S2: 50 candidate core KASP markers; Table S3: KASP result of 241 tomato accessions; Table S4: Genetic diversity analysis of 50 KASP markers in 241 tomato accessions; Table S5: 16 candidate core KASP markers; Table S6: 16 core germplasm typing results of 272 tomato accessions; Table S7: Comparison of Core/Key SNPs. Figure S1: KASP genotyping results; Figure S2: Cross Entropy Select Best K; Figure S3: Phylogenetic tree and Population structure of the 241 tomato accessions based on SNPs; Figure S4: Similarity heatmap of 272 tomato accessions; Figure S5: NJ tree unique fingerprint of 272 tomato accessions.
