# Supplementary material for: Development and Application of a KASP-Based SNP Marker System for Fingerprinting, and Genetic Diversity Analysis in Tomato
Source: Plants (Basel). 2026 Sep 26;15(19):2944. doi: 10.3390/plants15192944 (PMC13666781; doi:10.3390/plants15192944)
Supplement: Supplementary file 1 [file plants-15-02944-s001.zip › Supplementary Figures S1-S5.pdf]

Supplementary Figures:

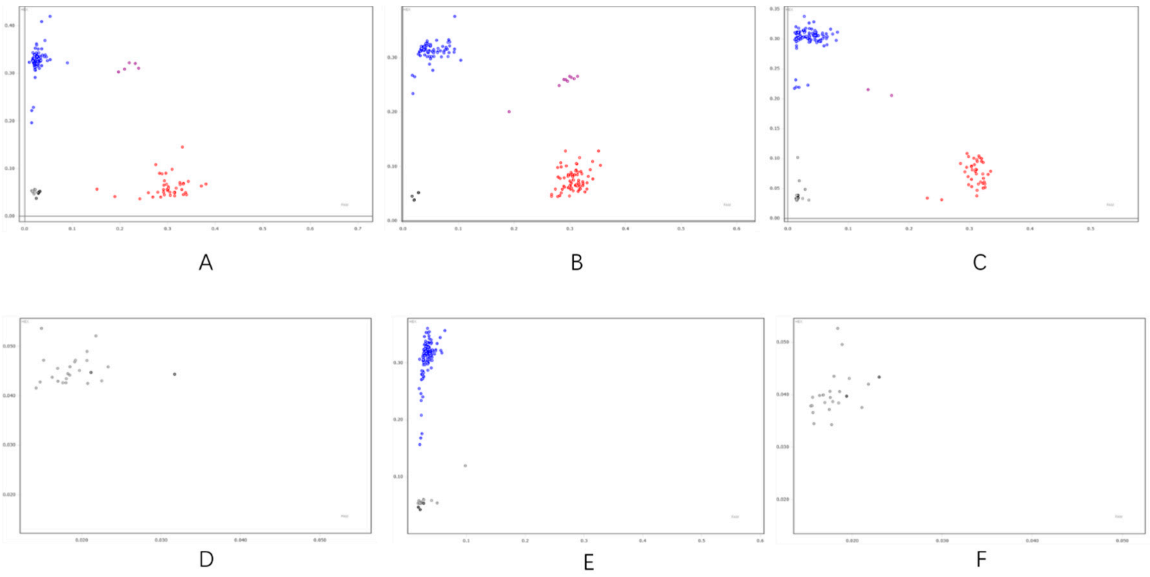

Supplementary Figure S1. KASP genotyping results

Fluorescence data were read and analyzed on the IntelliQube automated PCR system. The x- and y-axes represent the fluorescence signal intensities of FAM and HEX, respectively. Red fluorescence signals, matching the fluorescent tail of the forward primer F1, indicate that the sample carries the FAM signal. Blue fluorescence signals, matching the fluorescent tail of the forward primer F2, indicate that the sample carries the HEX signal. Black open circles represent the amplification signals of the no-template controls (NTCs), and gray open circles represent DNA samples in which no amplification signal was detected. Clusters in panels A–C passed quality criteria for downstream analysis, whereas those in panels D–F were excluded due to poor separation or excessive missing data. Notably, all 50 candidate core SNP no call rates were below 10% in 241 tomato materials. To assess genotyping reproducibility across plates, approximately 5% of samples were randomly selected and re-genotyped on separate plates. The inter-plate concordance rate was calculated as the percentage of samples with consistent genotype calls across plates. A concordance rate ≥ 98% was considered acceptable.

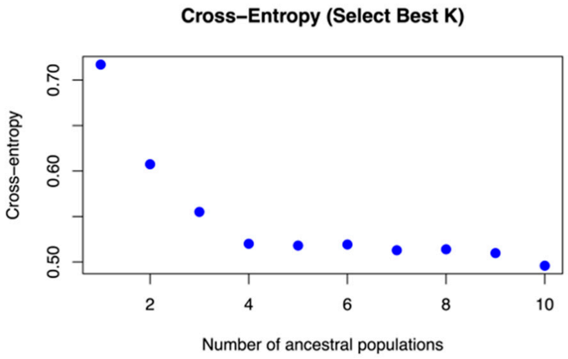

Supplementary Figure S2. Cross Entropy Select Best K

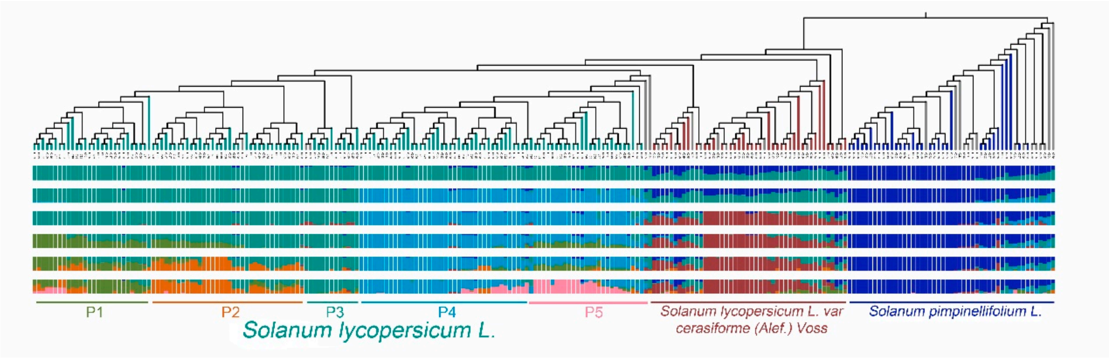

Supplementary Figure S3. Phylogenetic tree and Population structure of the 241 tomato accessions based on SNPs

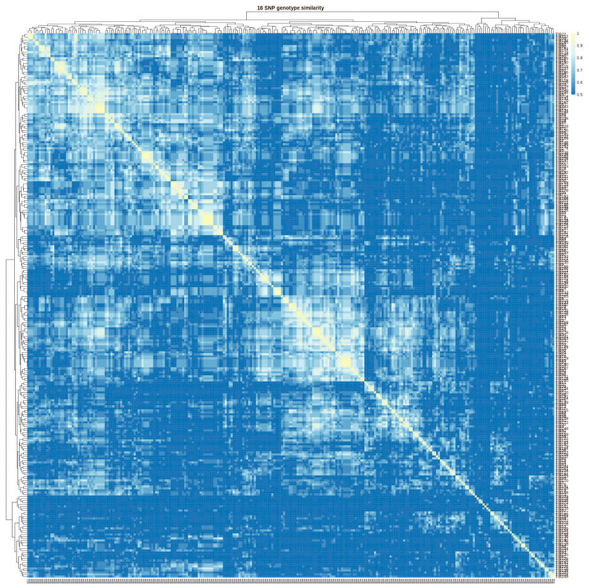

Supplementary Figure S4. Similarity heatmap of 272 tomato accessions

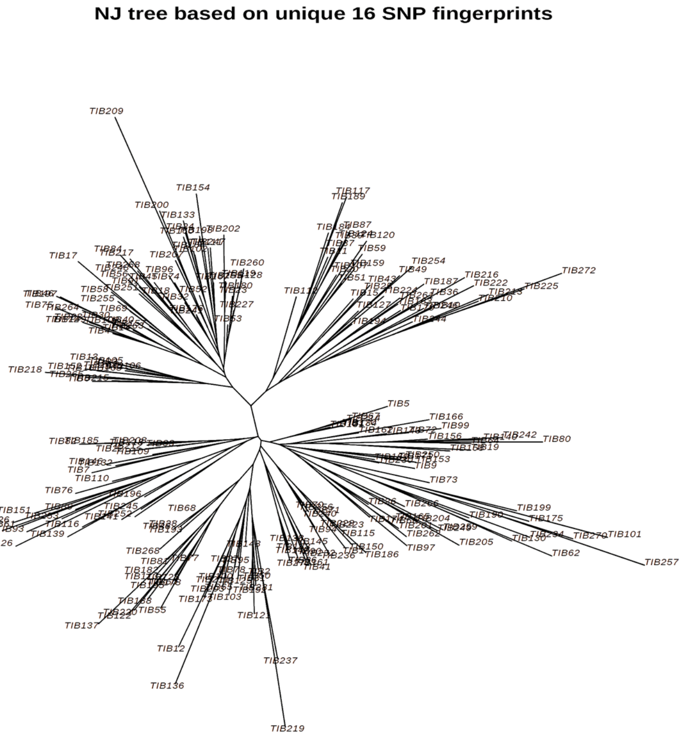

Supplementary Figure S5. NJ tree unique fingerprint of 272 tomato accessions
